# Safety assessment of poly-*ε*-caprolactone in the treatment of primary spontaneous pneumothorax

**DOI:** 10.3389/fsurg.2024.1335144

**Published:** 2024-01-19

**Authors:** Cheng-Hung How, Pei-Hsing Chen, Yu-Ching Chen, Yong-Chong Lin, Ke-Cheng Chen, Jin-Shing Chen, Tai-Horng Young

**Affiliations:** ^1^ Institute of Biomedical Engineering, College of Medicine and College of Engineering, National Taiwan University, Taipei City, Taiwan; ^2^ Department of Surgery, Division of Thoracic Surgery, Far Eastern Memorial Hospital, Taipei City, Taiwan; ^3^ Division of Thoracic Surgery, Department of Surgery, National Taiwan University Hospital Hsin-Chu Branch, Hsinchu, Taiwan; ^4^ Department of Surgery, National Taiwan University Hospital and National Taiwan University College of Medicine, Taipei City, Taiwan

**Keywords:** pleurodesis, pneumothorax, poly-*ε*-caprolactone, thoracoscopy, Phase I trial

## Abstract

**Background/purpose:**

Biomaterial-based implants are gaining traction as an option for pleurodesis treatment, yet the search for the best biomaterial or the most suitable shape to handle spontaneous pneumothorax continues. This forward-looking research assessed the use of a poly-*ε*-caprolactone membrane for its safety when applied as a sclerosant in pleurodesis procedures in human patients.

**Methods:**

From July 2017 to February 2018, we conducted a Phase I trial in which 10 patients with primary spontaneous pneumothorax were treated using video-assisted thoracoscopic surgery with a poly-*ε*-caprolactone membrane. These procedures encompassed bleb resection and mechanical pleurodesis through parietal pleura scrubbing. After resection, a 150 × 150 mm poly-*ε*-caprolactone membrane was applied to the apex. The primary outcome measures were the adverse events and laboratory outcomes.

**Results:**

After surgery, we observed no cardiopulmonary-related adverse events or indications of systemic inflammation. Furthermore, no episodes of hypothermia or hyperthermia occurred. Chest radiographs showed no evident pneumonitis or effusion associated with tissue reactions. The average follow-up duration was 31.7 ± 17.7 months, during which two patients exhibited recurrence.

**Conclusion:**

This study is the first to show the biocompatibility of poly-*ε*-caprolactone in humans, suggesting its potential as a treatment option for patients with primary spontaneous pneumothorax. Despite the relatively small number of patients, we maintain confidence in the reliability and safety profile of the PCL membrane, bolstered by its previously established efficacy in applications involving other organs. Phase II and phase III clinical studies are needed to support these observations.

## 1 Introduction

The management of spontaneous pneumothorax includes the cessation of air leak, elimination of pleural air, and prevention of recurrence (1). Surgical management is generally suggested for recurrent or complicated spontaneous pneumothorax; recent advancements in thoracic surgery, particularly in video-assisted thoracoscopic surgery (VATS) (2, 3), have established it as a forefront method for primary spontaneous pneumothorax treatment due to its minimally invasive nature (4). Yet, pneumothorax recurrence post-stapled bullectomy via VATS remains a concern, with rates spanning between 2% and 16% (5–7). This rate is influenced by the pleurodesis technique employed and is notably higher than the recurrence rate associated with open thoracotomy, which is reported to be less than 1% (8–10). The elevated postoperative recurrence observed with VATS can often result from overlooked leaking blebs proximate to the pleural staple line, coupled with a milder pleural inflammatory response elicited by VATS compared to thoracotomy. Further compounding this issue are instances of partial bleb resection or those performed adjacent to, or traversing, the lungs' emphysematous regions, leading to air leaks near the staple line (11, 12). Given this heightened recurrence risk, the effectiveness of simple bullectomy video-assisted thoracic surgery in PSP management has come under scrutiny.

Pleurodesis is considered an adjunctive therapy to surgery. Although many chemical sclerosants, including talc powder, minocycline, bleomycin, autologous blood patch, iodopovidone, picibanil and 50% dextrose, and silver nitrate, have been used to induce pleurodesis, all of which have drawbacks, especially related to pain sensation or unsatisfactory recurrence rates (13). Biomaterial implants are emerging as a treatment choice for pleurodesis; however, the ideal biomaterial or optimal form for the management of spontaneous pneumothorax is still under investigation (14–17). Several biomaterials have been reported for the treatment of repetitive catamenial pneumothorax (18, 19).

Previously, we demonstrated significant pleurodesis in New Zealand white rabbit animal models using poly-*ε*-caprolactone (PCL) membranes and found satisfactory results. However, the safety and efficacy of PCL membranes in humans remains unclear. Hence, this study aimed to determine the safety and/or adverse events of PCL membranes in a clinical phase I study.

## 2 Materials and methods

### 2.1 Participants and general design

This Phase I trial was conducted from July 2017 to February 2018 at National Taiwan University Hospital. The eligibility criteria were patients aged 15–50 years, with an indication for thoracoscopic bullectomy and pleurodesis, such as prolonged air leaks >3 days, ipsilateral pneumothorax recurrence, and contralateral pneumothorax. Written informed consent was obtained from all patients. Patients were included when they met the minimal requirement of hematological data (hemoglobin level > 10 g/dl, absolute neutrophil count > 1.5 × 10^3^/μl, and platelet count > 100 × 10^3^/μl). Patients were excluded if they had underlying chronic obstructive pulmonary disease, hemothorax, pneumothorax, catamenial pneumothorax, or other serious concomitant illnesses or malignancy or ongoing pregnancy.

This Phase I clinical trial was registered in the National Clinical Trial (clinicaltrials.gov number, NCT03227978) database and received ethics committee approval (201610065DINA).

### 2.2 PCL membrane

**Figure 1:**
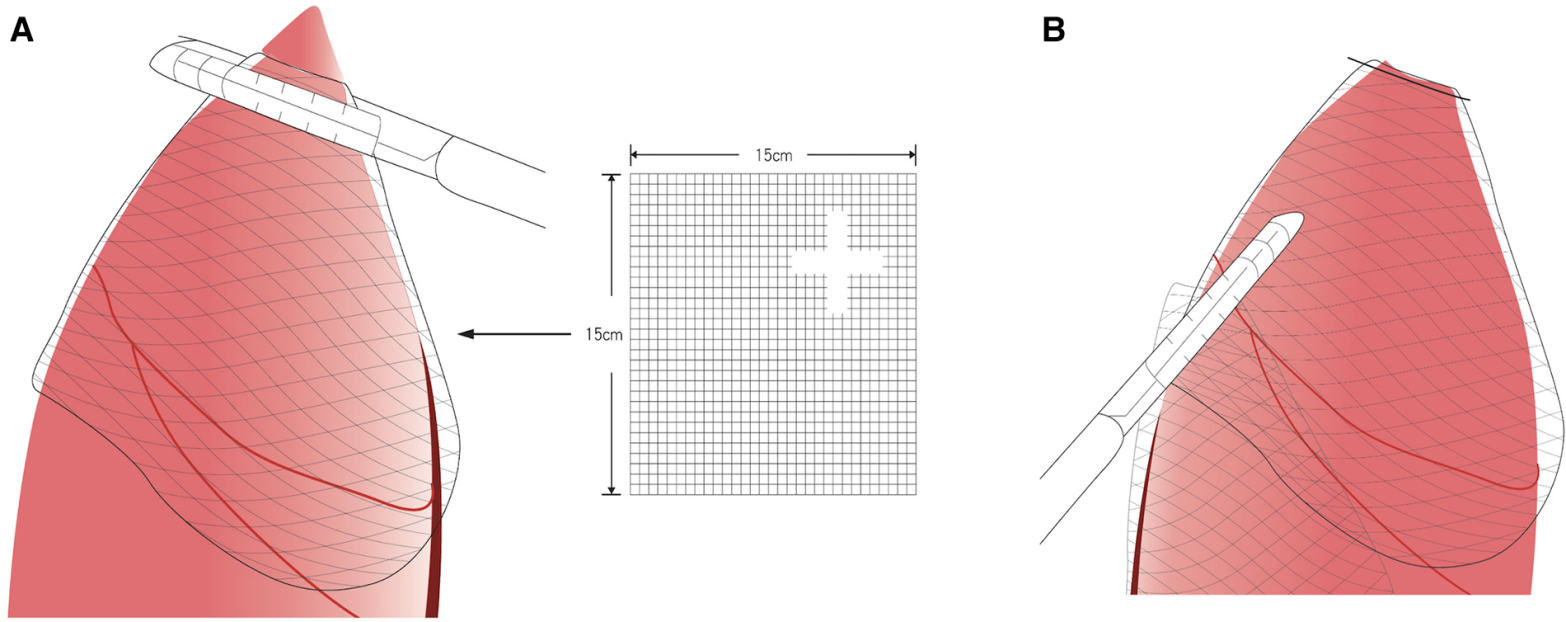
Poly-*ε*-caprolactone membrane fixed with the latest staple following the method by ikeda et al. (19) (**A**) Fix the membrane with the stapler (Illustration). (**B**) After stapler fixation (Illustration).

PCL (SigmaeAldrich, St. Louis, MO, USA) is bioresorbable polyester approved by the U.S. Food and Drug Administration and the European Union Food and Drug Administration. The sterile PCL membranes were fabricated using a solvent-casting method as described previously (20). The final membrane size was 150 × 150 mm (Figures 1A,B).

### 2.3 Surgical and anesthesia techniques

**Figure 2:**
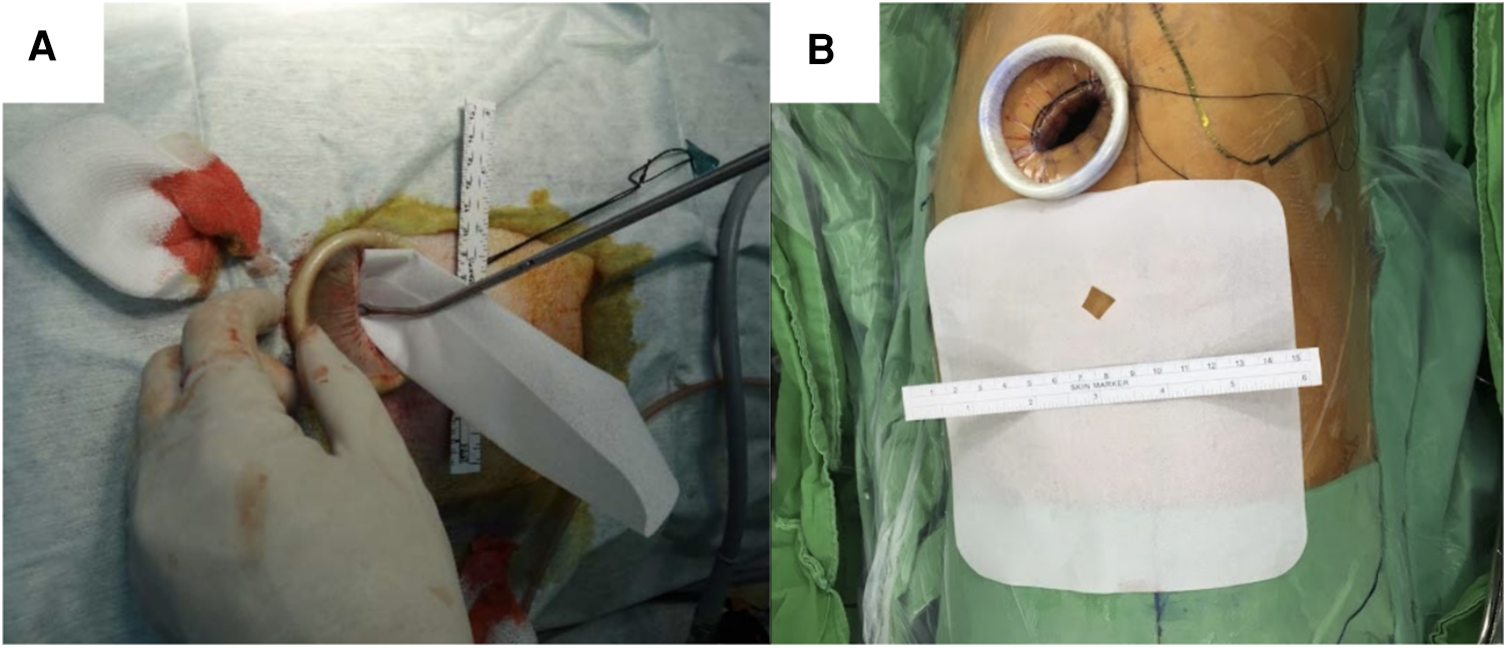
Poly-*ε*-caprolactone membrane (**A**) membrane structure. (**B**) Insert membrane in the chest cavity through the thoracoscopic wound.

The surgical and anesthesia techniques were performed as previously described (4, 16, 21). Briefly, all patients were intubated with a double-lumen endotracheal tube and placed in the lateral decubitus position. The ipsilateral lung was deflated during the procedure. The remaining steps of the thoracoscopy procedure was described in our previous study (14). A single skin incision (approximately 2.5–3 cm in length) was made at the fifth intercostal space at the middle axillary line (Figures 2A,2B). A vagus nerve block was performed to reduce the cough reflex, and an intercostal nerve block was used for pain control.

The lung was inspected for any possible bleb, and the adhesion was identified according to Vanderschueren's classification (22, 23). When a bleb was observed, an endoscopic stapler was introduced for resection. In cases of no or ruptured blebs, the resection was performed at the most suspicious area. Participants would undergo thoracoscopic bullectomy and pleural abrasion, then the PCL membrane (same material and size as used in the animal model experiments) was fixed with the latest staple following the method by Ikeda et al. (19) Thus, the membrane was sutured on the remaining lung (Figures 1A,B). The remaining procedure was identical to that previously described (24). After all the procedures were performed, a chest tube or pigtail was placed in the apex through the wound. The wound was closed after lung re-expansion. The bleb or apex lung was sent for pathological examination.

### 2.4 Adverse events and laboratory outcomes

The primary outcome of the clinical trial was abnormal arterial blood pressure or body temperature results. Laboratory data related to possible allergic responses, such as C-reactive protein levels, eosinophil levels, and white blood cell counts, were recorded. Data related to postoperative hospital stays were also analyzed.

Opioids were administered to patients every 4–6 h when the pain was not relieved by oral analgesics, or if the Visual Analogue Scale pain level score was >7. Chest radiography was routinely performed after surgery. The chest tube was removed after full lung expansion with no air leak in a 24-h period.

Post-operative complications were meticulously documented. Specifically, prolonged air leaks were characterized as air leaks persisting for a duration exceeding 5 days. Pleural detachment referred to the development of a pneumothorax immediately subsequent to the extraction of the chest tube.

### 2.5 Follow-up

**Figure 3:**
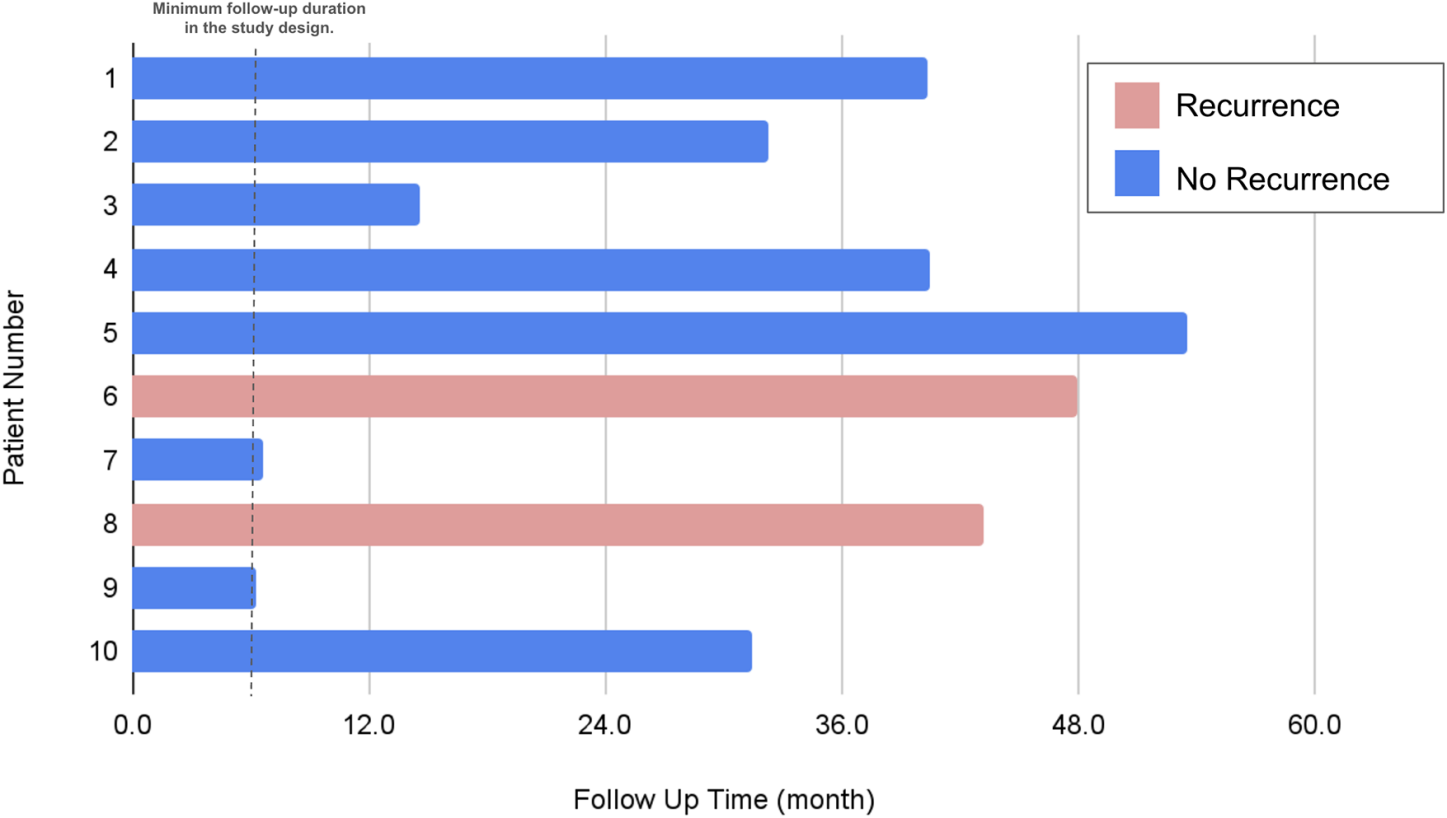
Evaluation of recurrence status. Of the two patients who experienced recurrence, both were taller than 170 cm and had a BMI below 20. The recurrence site for both was on the left side.

Following hospital discharge, patients were scheduled for outpatient clinic visits at intervals of 1 week, 1 month, 3 months, and 6 months. During each of these visits, chest radiography was conducted. All participants were suggested to be monitored for a minimum of 12 months. Furthermore, patients were advised to revisit the clinic or seek emergency care if they experienced symptoms such as chest pain, dyspnea, or any indications suggestive of a pneumothorax recurrence (Figure 3).

### 2.6 Statistical analyses

The demographic data and related surgical results, including operative findings, complications, and body weights, were recorded and analyzed statistically. Continuous variables, such as body weight and height, are expressed as mean ± standard deviation. Categorical variables, such as sex, are represented as frequency (%). Fisher's exact test was performed as a statistical significance test of contingency tables. Statistical analyses were performed using SPSS software (version 22.0; IBM Corp., Armonk, NY, USA), R (version 3.4.3; R Foundation for Statistical Computing, Vienna, Austria), and R Studio (version 1.1.414; RStudio, PBC, Boston, MA, USA).

## 3 Results

The extended investigation in the Phase I trial included a cohort of 10 patients, all diagnosed with primary spontaneous pneumothorax, who were administered treatments utilizing PCL membrane. Detailed patient demographics and clinical features are systematically cataloged in Table 1. The average age of the cohort was 27.6 years, with a standard deviation of 10.2 years, spanning from 20 to 46 years, and the majority of these patients (80%) were male. Stature and mass metrics averaged at 169.7 cm in height and 56.3 kg in weight, respectively. A significant outcome of the study was the absence of fatalities, coupled with a complete lack of necessity to escalate to multi-port VATS, as shown in Table 2. Blood loss was minimal across the board, with all patients losing less than 20 cc during surgery. The surgical intervention duration averaged at slightly over 54 min, with a variability of 18.5 min. Notably, none of the patients had a history of smoking.

**Table 1:** Patient characteristics.

	Membrane group (*N* = 10)
Age (years)^a^	27.6 ± 10.2
Male, *n* (%)	8.0 (80.0%)
Height (cm)^a^	169.7 ± 6.7
Weight (kg)^a^	56.3 ± 5.5
Body mass index^a^	19.6 ± 2.2
Side involved (left), *n* (%)	6 (60.0%)
Vanderschueren's classification	
Stage I: No abnormalities	1 (10.0%)
Stage II: pleuropulmonary adhesion	2 (20.0%)
Stage III:Blebs/bullae of <2 cm	5 (50.0%)
Stage VI:Bullae of >2 cm	2 (20.0%)
Number of bullae	
No bullae	3 (30.0%)
*N* = 1 or 2	6 (60.0%)
*N* > 2	1 (10.0%)

^a^ Mean ± standard deviation.

**Table 2:** Perioperative outcomes.

	*N* = 10
Operation time (minutes)	54.0 ± 18.5 (32–85)
Blood loss (ml)	Minimal^a^
Postoperative hospital admission (day)	4.3 ± 4.9 (2–18)
Postoperative prolonged air leak	1.0 (10%)
Chest tube dwelling day (days)	4.7 ± 7.2 (2–25)
Single-port method	10 (100%)
Follow-up time (months)	31.7 ± 17.7 (6.3–53.5)
Recurrence	2 (20%)

^a^ All patients experienced blood loss of less than 20 cc.

Following the surgery, the patients' recovery was notable for the lack of fever presentations. Furthermore, within the initial postoperative triad of days, there were no documented cases of temperature dysregulation, blood pressure abnormalities, or severe adverse reactions. Allergy assessments, indicated by vital signs and laboratory analysis, showed no significant hypersensitive responses. Inflammatory markers, such as C-reactive protein levels, and counts for white blood cells and eosinophils, were reported to be stable and within normal parameters (Table 3). Radiographic imaging post-procedure did not reveal any evidence of pneumonitis or effusions that could indicate a tissue reaction to the implant.

**Table 3:** Outcomes related to allergic response.

	*N* = 10
Hypothermia or Hyperthermia^a^	0 (0.0%)
Hypotension or Hypertension^b^	0 (0.0%)
Postoperative C-reactive protein (mg/L)	1.8 ± 1.2
Preoperative white blood count level	8.3 ± 0.3
Postoperative white blood count level	10.8 ± 3.4
Postoperative eosinophil ratio (%)	3.9 ± 2.2
Postoperative pneumonitis	0 (0.0%)
Postoperative pleural effusion	0 (0.0%)

^a^ Mean ± standard deviation.

^b^ With any record within 3 days after surgery.

The median hospitalization period post-procedure was 4 days, but this varied from as brief as 2 days to as extended as 18 days. Chest tubes were utilized in the vast majority (90%) of patients, typically remaining in place for a median of 3 days. One case stood out where a patient experienced prolonged air leakage for up to 25 days. This individual was discharged with a chest tube outfitted with a one-way valve system, which was later removed during a subsequent outpatient visit. The follow-up period for the study participants averaged at almost 32 months, with a spectrum ranging from half a year to just over three years, as depicted in Figure 3. Within this follow-up timeframe, two instances of pneumothorax recurrence were observed, as elaborated in Table 2. Notably, no perioperative complications were recorded. A commonality among patients who experienced a recurrence was a height exceeding 170 cm and a body mass index below 20, with recurrences exclusively occurring on the left side. At the start, these patients were all categorized as grade III under the Vanderschueren classification, which denotes the presence of blebs or bullae smaller than 2 cm.

## 4 Discussion

This research showed that the PCL membrane, when combined with bullectomy via VATS, is safe and effective for clinical pleurodesis procedures. No significant cardiopulmonary changes or adverse effects were observed in the clinical study, and there was no increase in inflammation indicators. The follow-up period remained uneventful.

Since pleurodesis is a possible treatment for primary pneumothorax, several studies have evaluated its effect in clinical situations (21, 24, 25). The most intensive pleurodesis agent may not always be the best choice due to the possibility of a severe inflammation response, pain sensation, or bleeding. The effect of a biomaterial with the conventional pleurodesis method, such as mechanical pleurodesis and bullectomy, remains uncertain (12, 22, 26).

Previous research demonstrated the PCL membrane's ability to create adhesive effects. It induced and enhanced tissue adhesion by changing the membrane from dense to porous tissue (27, 28). Our previous work also proved the adhesion ability, fibronectin level change, and fibrosis effects of PCL membrane in a rabbit model, and the PCL membrane showed potential in short-term results (30 days) (14, 20).

While its biocompatibility has been clinically evaluated for safety in human participants, the recurrence rate in this trial appears to be higher than in previous studies (21, 25, 29), even when compared to simple pleural abrasion (18). While the recurrence rate of pneumothorax after thoracoscopic surgery typically ranges from 2% to 14% according to previous studies (30), our study observed two patients experiencing recurrence. However, it is essential to note that the primary objective of this trial was to evaluate safety. In our routine clinical practice, as described in our recently published work (4), we employ a specific method: we use a woven absorbable mesh (Vicryl mesh, 30 cm × 30 cm). In our previously published technique, the mesh is introduced into the pleural cavity through an access port, and we meticulously position it on the apical visceral pleura to ensure coverage of the staple line, adhering to the method of Ikeda et al. (19). Furthermore, some cases were performed using a non-intubated method, representing a surgical innovation (31, 32).

However, in this study, we employed only a quarter of the typical mesh size, primarily as a safety precaution, which differs from the methodologies in previous studies (4, 19). This decision to use a reduced mesh size might be the reason for the observed recurrence in patients taller than 170 cm. We expect the recurrence rate to be lower in future phase II or phase III trials when using the mesh at its intended size. In our study, we observed a patient with a prolonged air leak that persisted for 25 days. Utilizing a larger size of the PCL membrane for coverage could potentially have ameliorated this situation. Furthermore, no postoperative adverse events were observed in the human participants. This suggests that the PCL membrane is both safe and reliable as a biomaterial for treating patients with primary spontaneous pneumothorax.

Limitations of this study include the small number of patients in the clinical trial. Further, the clinical trial demonstrated only the PCL membrane's safety, but not its effectiveness; two patients experienced recurrence during the follow-up period. Further studies (Phase II and Phase III) are required to establish the effectiveness of the PCL membrane for patients with primary pneumothorax. Despite the relatively small number of patients, we maintain confidence in the reliability and safety profile of the PCL membrane, bolstered by its previously established efficacy in applications involving other organs.

In conclusion, this study is the first to report the safe use of PCL in humans when utilized in combination with VATS bullectomy. Our results demonstrate the PCL membrane's potential for treating patients with primary spontaneous pneumothorax.

## Data Availability

The raw data supporting the conclusions of this article will be made available by the authors, without undue reservation.
