# Corrigendum: Safety assessment of poly-*ε*-caprolactone in the treatment of primary spontaneous pneumothorax

**DOI:** 10.3389/fsurg.2024.1386747

**Published:** 2024-02-29

**Authors:** Cheng-Hung How, Pei-Hsing Chen, Yu-Ching Chen, Yong-Chong Lin, Ke-Cheng Chen, Jin-Shing Chen, Tai-Horng Young

**Affiliations:** ^1^ Institute of Biomedical Engineering, College of Medicine and College of Engineering, National Taiwan University, Taipei City, Taiwan; ^2^ Department of Surgery, Division of Thoracic Surgery, Far Eastern Memorial Hospital, Taipei City, Taiwan; ^3^ Division of Thoracic Surgery, Department of Surgery, National Taiwan University Hospital Hsin-Chu Branch, Hsinchu, Taiwan; ^4^ Department of Surgery, National Taiwan University Hospital and National Taiwan University College of Medicine, Taipei City, Taiwan

**Keywords:** pleurodesis, pneumothorax, poly-*ε*-caprolactone, thoracoscopy, Phase I trial

A Corrigendum on Safety assessment of poly-*ε*-caprolactone in the treatment of primary spontaneous pneumothorax By How C-H, Chen P-H, Chen Y-C, Lin Y-C, Chen K-C, Chen J-S and Young T-H (2024). Front. Surg. 11:1335144. doi: 10.3389/fsurg.2024.1335144

**Incorrect Funding**

In the published article, there was an error in the Funding statement. [The author acknowledges the support from the National Science and Technology Council project, Grant No. 112-2314-B-002-226-MY3. This research was jointly supported by the Far Eastern Memorial Hospital and the National Taiwan University Hospital Joint Research Program.]. The correct Funding statement appears below.

